# Environmental variables associated with anopheline larvae distribution and abundance in Yanomami villages within unaltered areas of the Brazilian Amazon

**DOI:** 10.1186/s13071-017-2517-6

**Published:** 2017-11-16

**Authors:** Jordi Sánchez-Ribas, Joseli Oliveira-Ferreira, John E. Gimnig, Cleomar Pereira-Ribeiro, Maycon Sebastião Alberto Santos-Neves, Teresa Fernandes Silva-do-Nascimento

**Affiliations:** 1 Laboratório de Imunoparasitologia, Instituto Oswaldo Cruz-FIOCRUZ, Rio de Janeiro, Brazil; 2 Laboratório de Mosquitos Transmissores de Hematozoários, Instituto Oswaldo Cruz-FIOCRUZ, Rio de Janeiro, Brazil; 3 Center for Disease Control and Prevention, CDC, Atlanta, USA; 4 Distrito Sanitário Especial Indígena Yanomami, DSEI-Y, Boa Vista, Brazil

**Keywords:** Anopheline larvae, Environmental drivers, Sun exposure, *An. darlingi*, Yanomami

## Abstract

**Background:**

Many indigenous villages in the Amazon basin still suffer from a high malaria burden. Despite this health situation, there are few studies on the bionomics of anopheline larvae in such areas. This publication aims to identify the main larval habitats of the most abundant anopheline species and to assess their associations with some environmental factors.

**Methods:**

We conducted a 19-month longitudinal study from January 2013 to July 2014, sampling anopheline larvae in two indigenous Yanomami communities, comprised of four villages each. All natural larval habitats were surveyed every two months with a 350 ml manual dipper, following a standardized larval sampling methodology. In a third study area, we conducted two field expeditions in 2013 followed by four systematic collections during the long dry season of 2014–2015.

**Results:**

We identified 177 larval habitats in the three study areas, from which 9122 larvae belonging to 13 species were collected. Although species abundance differed between villages, *An. oswaldoi* (*s.l*.) was overall the most abundant species. *Anopheles darlingi*, *An. oswaldoi* (*s.l*.), *An. triannulatus* (*s.s*.) and *An. mattogrossensis* were primarily found in larval habitats that were partially or mostly sun-exposed. In contrast, *An. costai*-like and *An. guarao*-like mosquitoes were found in more shaded aquatic habitats. *Anopheles darlingi* was significantly associated with proximity to human habitations and larval habitats associated with river flood pulses and clear water.

**Conclusions:**

This study of anopheline larvae in the Brazilian Yanomami area detected high heterogeneities at micro-scale levels regarding species occurrence and densities. Sun exposure was a major modulator of anopheline occurrence, particularly for *An. darlingi*. Lakes associated with the rivers, and particularly oxbow lakes, were the main larval habitats for *An. darlingi* and other secondary malaria vectors. The results of this study will serve as a basis to plan larval source management activities in remote indigenous communities of the Amazon, particularly for those located within low-order river-floodplain systems.

**Electronic supplementary material:**

The online version of this article (10.1186/s13071-017-2517-6) contains supplementary material, which is available to authorized users.

## Background

Malaria is a preventable and treatable parasitic disease but still poses a high burden in some countries of Latin America, particularly Brazil which was responsible for 24% of the total number of cases in 2015 [1]. Disease distribution is heterogeneous within endemic regions in the Neotropics, and some populations are at high risk of acquiring malaria, such as indigenous communities in the Amazon basin and Central America [2, 3]. A prime example is the Yanomami people, considered the largest, semi-isolated indigenous group that inhabits a 192,000 km^2^ area split between Brazil and Venezuela. Malaria incidence is unevenly distributed across the Brazilian Yanomami Indian Reserve with some areas with very reduced malaria receptivity and other localities that are hotspots of intense malaria transmission, including for *Plasmodium falciparum* [4]. Hotspots in indigenous areas should be priority target areas for malaria elimination in Latin America, as in addition to the severe malaria burden on the indigenous populations, they may serve as a continuous source of *Plasmodium* spp. infections for neighbouring non-indigenous areas.

In line with the current global drive for malaria elimination, most Latin American countries have adopted country or sub-regional strategies for the elimination of *Plasmodium* spp*.* transmission [5]. In November 2015, Brazil launched its plan for countrywide malaria elimination, with a first phase focusing on sustainably eliminating *P. falciparum* transmission [6]. In Brazil, malaria control is mainly based on early diagnosis and treatment and adult vector control strategies such as indoor residual spraying (IRS) and long-lasting insecticidal nets (LLINs) [2]. These strategies, which target primarily endophagic and endophilic mosquitoes, may have limited effectiveness against Neotropical malaria vectors which frequently feed and rest outdoors. Consequently, classical anti-adult measures may only partially suppress transmission due to *Anopheles darlingi*, the main Amazon malaria vector [7]. Thus, additional vector control tools are required to help to further reduce malaria in Latin America. Novel vector control strategies to tackle outdoor transmission include long-lasting insecticidal hammocks (LLIH) for forest and mobile populations who may sleep outdoors [7, 8], topical repellents for personal protection [9] and spatial repellents [10]. All these strategies aim to reduce the contact between humans and exophagic host-seeking malaria vectors and in the case of LLIH, they may also minimize indoor exposure to mosquito bites in indigenous dwellings [7].

Also, there is a renewed interest in strategies targeting anophelines during their most vulnerable aquatic phase. This approach has been indicated when larval habitats are few, fix and findable [11]. However, to apply larval source management (LSM) activities efficiently, a comprehensive knowledge of the local ecology of anopheline larvae and their aquatic habitats is required. This has been considered a major drawback in implementing LSM activities in many settings [12]. Nevertheless, LSM is a potential tool to reduce or eliminate anopheline populations insufficiently controlled by IRS and LLINs in some malaria-endemic localities of the Neotropical region.

Comprehensive information on malaria epidemiology and vector bionomics will be necessary to eliminate malaria, particularly in these remote and hard-to-work indigenous areas. The variability in malaria risk in the Neotropics and within Indian Reserves is due to a complex interaction of many ecological and social determinants of the disease. Among the factors influencing transmission, entomological parameters of a few highly competent malaria vectors such as *An. darlingi* and *An. albimanus* are of major importance [13]. The spatio-temporal distribution of anopheline larvae and thus, adult host-seeking and pathogen transmitting anophelines depend on parameters such as the number, quality and size of potential larval habitats, their distance from blood meal sources and a wide range of other environmental factors [14].

Few entomological investigations have been conducted in nearby areas of the Brazilian Yanomami Indian Reserve. Suarez-Mutis et al. [15] and Hutchings et al. [16] focused on sampling adult mosquitoes in the Padauari river, Amazon State, and Cabral et al. [17] reported both adult and larval collections. Also in Brazil, a larval study was conducted east of the Indian Reserve [18] while another report summarized the adult anopheline occurrence in multiple collecting points within Roraima State [19]. There have been several entomological reports focusing on adults [20–22] as well as larvae [23–25] in neighbouring areas of Venezuela. In this publication, we provide the first detailed anopheline species inventory, identify the larval habitat preferences and analyze some environmental drivers that modulate anopheline occurrence and densities in remote Yanomami indigenous communities of the Brazilian Amazon. We report results concerning larval habitats for seven anopheline species (six species of the genus *Anopheles* plus *Chagasia bonneae*), including the main malaria vector of the Amazon rainforest, *An. darlingi*. This information will help to devise feasible, sustainable and cost-effective LSM activities, targeting mainly *An. darlingi* immature forms in resilient transmission hotspots of the Yanomami endemic area, primarily within low-order Amazonian river-floodplain systems.

## Methods

### Study area

**Fig. 1:**
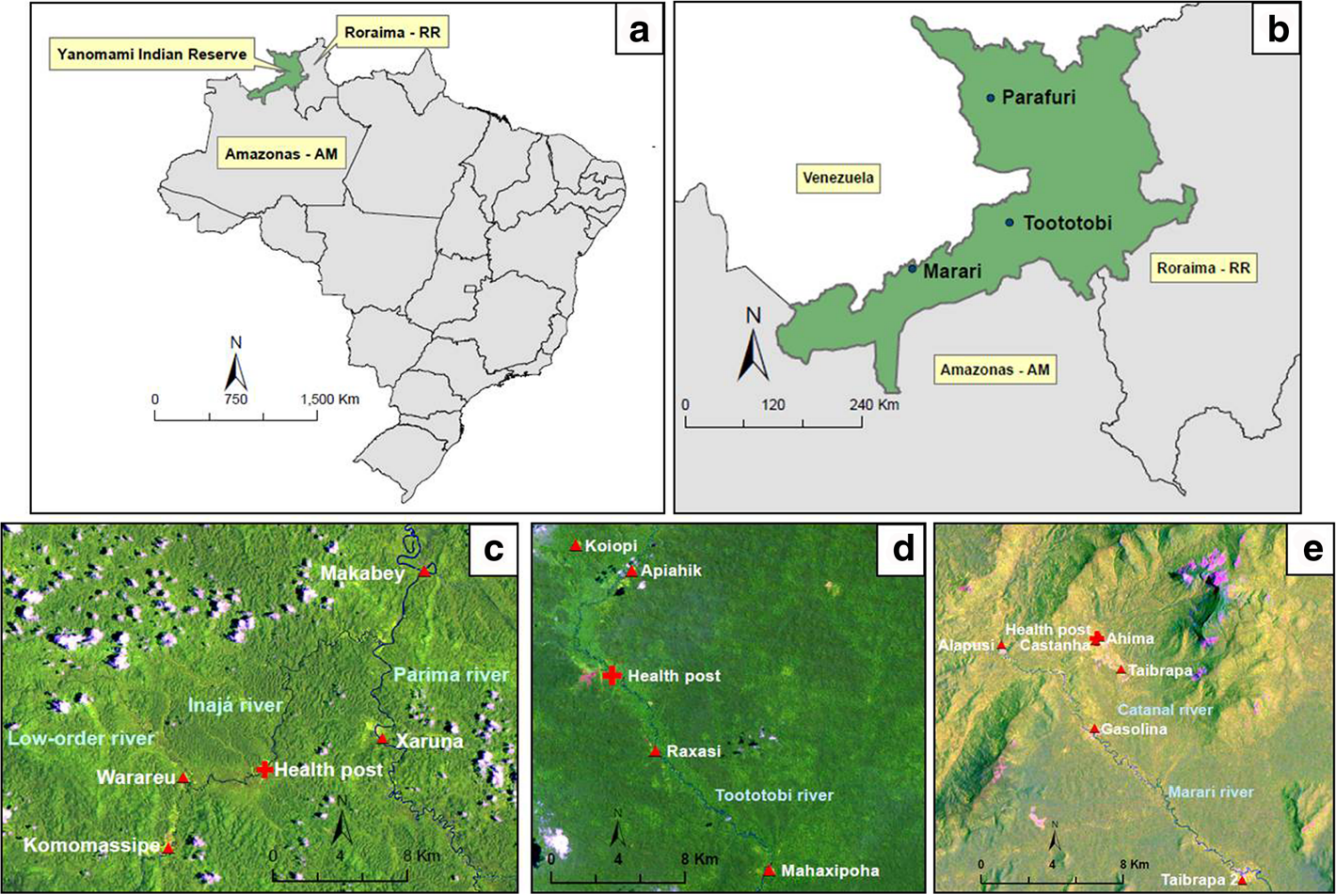
**a** Map of Brazil showing the location of the Yanomami Indian Reserve (green area). **b** The three Yanomami communities within the Reserve boundaries. **c**, **d** and **e** Detail of the villages and health posts of Parafuri (Roraima state, RR), Toototobi (Amazonas state, AM) and Marari (Amazonas state, AM) communities, respectively. The visible low-order rivers of the regions are highlighted. Source for LANDSAT imaginary: PRODESS 2009, INPE

We performed our study in three remote Yanomami communities in the northernmost region of the Brazilian Amazon, namely Parafuri, Toototobi and Marari (Fig. 1). In Parafuri and Toototobi communities, bimonthly collections were performed during 19 months, from January 2013 to July 2014, a time when only sporadic malaria cases were reported. A detailed description of these areas has been provided elsewhere [26]. Briefly, Parafuri is a hilly Amazonian sub-montane forested area with altitude 440 m above sea level (masl), and Toototobi community is located in a low-land Amazonian rainforest (128 masl). In each field expedition, we spent 15 days in each Yanomami community and concentrated our samplings in the same four villages in each community.

The third Yanomami community, Marari, is in a lowland Amazonian rainforest area (139 masl), which is drained by first to third order rivers and surrounded by nearby high mountains. The sampling efforts in the Marari community consisted of two pilot studies in 2013 and four field collections that covered the long dry season, from September 2014 to March 2015. In Marari, there is perennial and periodically intense malaria transmission, and the community is characterized by villages with high population density and a high risk of year-round immigration of parasite carriers from highly endemic areas outside the Yanomami Indian Reserve.

### Larval habitat definition and sampling strategy

We used a defined set of criteria to classify the natural larval habitats that we encountered in these Amazonian low-order river-floodplain systems. In short, association with river flood pulses, seasonality and degree of sun exposure were the main characteristics for larval habitat classification. Lakes associated with the river (LAR), which were subdivided into oxbow lakes (OX) and non-oxbow lakes (NOX), were permanent and always associated with river flood pulses. These larval habitats had a high degree of sun exposure. Flooded areas associated with the river (FAAR), which are also associated with river water levels fluctuations, were always seasonal. These larval habitats varied in their degree of sun exposure. We also identified inland water bodies which were out of the reach of river flood pulses and formed mostly due to increased rainfall, such as flooded areas not associated with the river (FANAR), rainfall pools (RP) and small (SFS) and medium forest streams (MFS). These aquatic habitats not associated with river flood pulses were predominantly shaded.

We identified all larval habitats within a 1 km radius of each village with the help of a local guide. Larval habitats were sampled following a standardized methodology. We used a fine-scale laser rangefinder (Scout DX 1000 ARC, Bushnell®, Overland Park, USA) to accurately quantify the perimeter of all larval habitats and after adjusting for the presence of additional niches adequate for larval proliferation, we estimated the total effective breeding area (tEBA) for each larval habitat. We defined the tEBA as the sum of all those portions of the water body surface that were suitable for anopheline proliferation. Based on the tEBA, we conducted some dips for each larval habitat. Dips were taken from the different EBA subtypes of all larval habitats. For larger larval habitats, a small portable inflatable boat was used to collect larvae that could not be reached from the edge of the habitat. Each larval habitat constituted a single record comprised of the larval collections of different subtypes of EBA. We recorded the total number of anophelines per dip, and then transferred larvae from standard 350 ml dippers (BioQuip, Rancho Dominguez, CA, USA) to plastic tubes with 80% ethanol. We reared a subset of field-collected L4 larvae in individual vials to obtain larval and pupal skins and their associated adult forms. A more detailed description of the classification of natural breeding habitats within low-order river-floodplain systems, procedures for the location of larval habitats and the larval sampling methodology used in our study area have been provided elsewhere [26]. We employed MosqTent traps [27] to collect host-seeking adult female anophelines during three or four consecutive nights in each village, alternating during each field trip between 4 h (18:00–22:00 h) collections at the same time in the intra, peri and extradomicilliary environments and 12 h collections in the peridomicily or extradomicily. These adult collections occurred concomitantly with the larval samplings. Larvae (third and fourth-instar) and adults were identified using the keys of Consoli and Lourenço-de-Oliveira and Forattini [28, 29].

### Assessment of environmental variables

Larval habitats were geo-referenced with a hand-held global positioning system (GPS) device. We recorded environmental variables for each aquatic habitat during each field survey, including its association with river flood pulses, seasonality (seasonal or permanent), sun exposure (shaded, partially sun-exposed or mostly sun-exposed), presence of submersed macrophytes, distance to the nearest human habitation, water turbidity (clear, semi-turbid or turbid) and water movement (stagnant waters or with some water movement). Permanent aquatic habitats were those that had water in all our field visits, while temporary ones were those that were completely dry on at least one visit. Sun exposure for each habitat was categorized as (i) mostly sun-exposed sites if between 75 and 100% of the tEBA of the larval habitat was exposed to the sun for a reasonable amount of time; (ii) partially exposed to the sun if between 25 and 75% of the tEBA was exposed to sunlight; and (iii) shaded if less than 25% of the tEBA was exposed to direct sunlight. Typically, water bodies of this last category were under dense forest cover, and little or no sunlight reached their tEBA. The distance from each larval habitat to the nearest dwelling was calculated using the BaseCamp software (Garmin, Olathe, KS, USA). Turbidity was determined by collecting a small sample from the water surface layer with a crystal pot and determining the visibility of two differential density black lines drawn on a white paper and placed on the far side of the pot. If both lines were visible, only the thickest or none of them, we classified water turbidity as clear, semi-turbid or turbid, respectively. Lastly, larval habitats classified with water movement presented some degree of current (longitudinal water movement) and some degree of constant water levels fluctuations due to its connection with a pulsing-system, such as riverbed pools or LAR directly connected to the river.

### Data analysis

We combined data for each species from the three communities to obtain a wider picture of anopheline ecological parameters within the Brazilian Yanomami Indian Reserve. All three areas are drained by low-order and clear water rivers, presented the same type of larval habitats and many species were common in the three areas. Separate analyses were done for each species that could be identified. We did not conduct any analyses on anopheline mosquitoes which we were unable to identify to species. All descriptive statistics were adjusted to estimate the number of larvae per 100 dips.

We analyzed data using a negative binomial regression, which uses counts as the outcome variable and is appropriate for overdispersed data where many counts are zeros. We considered as a dependent variable the number of larvae adjusted for the number of dips, which worked as the model offset, correcting for the differences in the number of dips per larval habitat, which depended on the tEBA calculation per larval habitat in each survey [26]. For *An. darlingi*, the outcome included the total number of all instars as L1-, and L2-instars of these species could easily be identified. For all other species, only the total number of L3- and L4-instars were included in analyses. We included in our final dataset a total of 711 data entries for analysis. Visits during which larval habitats were dry were excluded from the analysis, but the information was used for determining the seasonality of the habitat. We conducted univariate analyses for seven anopheline species from which we retrieved sufficient specimens and explored the effect of each environmental variable on mosquito densities independently. We then considered in multivariate regression models a continuous variable (distance from larval habitats to nearest Yanomami dwelling) and up to 6 categorical variables (association with flood pulses, seasonality, the degree of sun exposure, turbidity, water movement and presence of submersed macrophytes). As we detected strong effect modification between variables in the adjusted model for *An. triannulatus* (*s.s*.), an interaction term between seasonality and sun exposure was included in the analysis to obtain reliable outcomes. For *An. triannulatus* (*s.s*.) and *Chagasia bonneae*, only five variables were considered in the multivariate model as two variables had categories with no mosquitoes collected. Those comparisons with *P* < 0.05 were considered statistically significant.

We entered the data into a database created using the Epi Info software (Epi Info™, Atlanta, GA, USA). For processing geo-data and LANDSAT images, we used ArcGIS software (ESRI, Redlands, CA, USA). Data management and statistical analyses were done using SAS software (SAS Institute Inc., Cary, NC, USA).

## Results

### Descriptive data

We collected a total of 9122 anopheline larvae (including all *Anopheles* spp. and *Chagasia bonneae*) from a total of 177 larval habitats, from where we performed 71,288 dips over the entire study period. We identified a total of 13 species, four species of the subgenus *Nyssorhynchus* [*An. darlingi*, *An. oswaldoi* (*s.l*.), *An. triannulatus* (*s.s*.) and *An. nuneztovari* (*s.l*.)], four species of the subgenus *Anopheles* (*An. mattogrossensis*, *An. intermedius*, *An. guarao*-like and *An. costai*-like), three species of subgenus *Stethomyia* (*An. kompi*, *An. thomasi* and *An. nimbus*), one member belonging to the *Lophopodomyia* subgenus (*An. squamifemur*) and one species of the genus *Chagasia* (*Chagasia bonneae*). Hereafter, we focus on the seven most abundant species collected.

We identified a total of 1966 (21.6%) late-instar larvae (third and fourth stage) to species level. We were unable to identify at species level 5979 (65.5%) larvae (1970, 1949 and 2060 unidentified larvae in Toototobi, Parafuri and Marari, respectively), mainly because they were early instar (first and second stages) larvae and they were not *An. darlingi*. We also identified 1177 (12.9%) early instar *An. darlingi* larvae. The results of larvae collections stratified per Yanomami community are shown in Table 1. The results stratified by village are provided in Additional file 1: Table S1.

**Table 1:** Diversity and number (percentage) of *Anopheles* species collected per Yanommami community. We have not included the 1177 early instar *An. darlingi* larvae identified in the table. The number of early instar *An. darlingi* for each community was as follows; Toototobi (2), Parafuri (993) and Marari (182)

Species	Toototobi	Parafuri	Marari	Total
*An. darlingi*	0	407 (53.3)	96 (18.0)	503 (25.6)
*An. oswaldoi* (*s.l*.)	321 (47.8)	81 (10.6)	218 (41.0)	620 (31.5)
*An. triannulatus* (*s.s*.)	38 (5.7)	163 (21.4)	8 (1.5)	209 (10.6)
*An. guarao*-like	104 (15.5)	37 (4.8)	28 (5.3)	169 (8.6)
*An. costai*-like	87 (13.0)	50 (6.6)	28 (5.3)	165 (8.4)
*An. mattogrossensis*	104 (15.5)	0	34 (6.4)	138 (7.0)
*An. nuneztovari* (*s.l*.)	0	0	79 (14.8)	79 (4.0)
*An. intermedius*	3 (0.4)	0	12 (2.3)	15 (0.8)
*An. nimbus*	3 (0.4)	2 (0.3)	9 (1.7)	14 (0.7)
*An. squamifemur*	0	7 (0.9)	0	7 (0.4)
*An. kompi*	4 (0.6)	1 (0.1)	0	5 (0.3)
*An. thomasi*	0	1 (0.1)	0	1 (0.1)
*Chagasia bonneae*	7 (1.0)	14 (1.8)	20 (3.8)	41 (2.1)
Total	671	763	532	1966

We identified to species level 671 mosquitoes in Toototobi community. *Anopheles oswaldoi* (*s.l*.) (47.8%) was the most widely disseminated species and was the most abundant in three out of four villages, followed in overall counts by *An. mattogrossensis* (15.5%) and *An. guarao*-like (15.5%). In contrast, *An. darlingi* was the predominant species (53.3%) among the 763 anophelines identified in the hilly submontane rainforest area of Parafuri community, followed by *An. triannulatus* (*s.s*.) (21.4%) and *An. oswaldoi* (*s.l*.) (10.6%). *Anopheles darlingi* abundance was markedly heterogeneous amongst Parafuri community. One village accounted for 97.1% of all *An. darlingi* collections within Parafuri community, while this species was absent in two villages and collected in low densities in another (4.9%) and the vicinities of the Health Post (3.5%). In the third Yanomami community of Marari, we identified to species level 532 anophelines. *Anopheles oswaldoi* (*s.l*.) was again the predominant species (41.0%), followed by *An. darling* (18.0%)*.* However, the *An. darlingi*/*An. oswaldoi* (*s.l*.) the ratio was heterogenous between villages. *Anopheles triannulatus* (*s.s*.) and *An. nuneztovari* (*s.l*.) were only collected in one village, the latter being found in very high densities.

The total of anophelines collected in each type of larval habitat per community is shown in Table 2. We found substantial variability in the productivity of *An. darlingi* within OX and NOX between communities and villages. For example, only two L2 larvae of *An. darlingi* were collected during 19 months of sampling efforts in one shaded OX of Toototobi community. In contrast, a single mostly sun-exposed OX in Parafuri community accounted for 78.8% of *An. darlingi* (all instars) collections of this community. In Marari, *An. darlingi* was the second most abundant species collected in OX after *An. oswaldoi* (*s.l*.). Overall, *An. darlingi* was primarily found in the OX hydrological type with 67.7% of this species collected in OX.

**Table 2:** Diversity and number (percentage) of late instar anopheline larvae (L3 and L4 only) collected per type of larval habitat and Yanomami community

Community/species	OX	NOX	FAAR	FANAR	RP	SFS	MFS	RIV	Total
Toototobi	*n* = 8	*n* = 2	*n* = 11	*n* = 20	*n* = 1	*n* = 6	*n* = 8	*n* = 3	
*An. darlingi*	0	0	0	0	0	0	0	0	0
*An. oswaldoi* (*s.l*.)	70 (39.1)	0	86 (48.0)	77 (45.3)	0	28 (49.1)	60 (71.4)	0	321 (47.8)
*An. mattogrossensis*	49 (27.4)	1 (100.0)	46 (25.7)	5 (2.9)	0	0	3 (3.6)	0	104 (15.5)
*An. guarao-*like	36 (20.1)	0	24 (13.4)	26 (15.3)	0	3 (5.2)	15 (17.9)	0	104 (15.5)
*An. costai-*like	1 (0.6)	0	4 (2.2)	59 (34.7)	1 (100.0)	17 (29.8)	5 (6.0)	0	87 (13.0)
*An. triannulatus* (*s.s*.)	23 (12.8)	0	15 (8.4)	0	0	0	0	0	38 (5.7)
*Chagasia bonneae*	0	0	1 (0.6)	0	0	5 (8.8)	1 (1.2)	0	7 (1.0)
Other anophelines	0	0	3 (1.7)	3 (1.8)	0	4 (7.0)	0	0	10 (1.4)
Subtotal	179	1	179	170	1	57	84	0	671
Parafuri	*n* = 10	*n* = 9	*n* = 4	*n* = 10	*n* = 2	*n* = 11	*n* = 7	*n* = 5	
*An. darlingi*	318 (58.5)	80 (72.7)	0	1 (2.2)	8 (66.7)	0	0	0	407 (53.3)
*An. oswaldoi* (*s.l*.)	62 (11.4)	8 (7.3)	7 (58.3)	4 (8.9)	0	0	0	0	81 (10.6)
*An. mattogrossensis*	0	0	0	0	0	0	0	0	0
*An. guarao-*like	0	17 (15.5)	4 (33.3)	9 (20.0)	4 (33.3)	3 (11.1)	0	0	37 (4.8)
*An. costai-*like	4 (0.7)	1 (0.9)	0	28 (62.2)	0	5 (18.5)	12 (92.3)	0	50 (6.6)
*An. triannulatus* (*s.s*.)	160 (29.4)	3 (2.7)	0	0	0	0	0	0	163 (21.4)
*Chagasia bonneae*	0	1 (0.9)	1 (8.3)	0	0	11 (40.7)	1 (7.7)	0	14 (1.8)
Other anophelines	0	0	0	3 (6.6)	0	8 (29.6)	0	0	11 (1.4)
Subtotal	544	110	12	45	12	27	13	0	763
Marari	*n* = 16	*n* = 6	*n* = 6	*n* = 11	*n* = 1	*n* = 7	*n* = 5	*n* = 8	
*An. darlingi*	65 (22.0)	12 (12.4)	11 (44.0)	0	0	4 (6.3)	0	4 (25.0)	96 (18.0)
*An. oswaldoi* (*s.l*.)	96 (32.5)	77 (79.4)	1 (4.0)	4 (13.8)	0	32 (50.0)	1 (16.7)	7 (43.8)	218 (41.0)
*An. mattogrossensis*	26 (8.8)	7 (7.2)	1 (4.0)	0	0	0	0	0	34 (6.4)
*An. guarao-*like	18 (6.1)	0	1 (4.0)	7 (24.1)	0	1 (1.6)	0	1 (6.3)	28 (5.3)
*An. costai-*like	2 (0.7)	0	0	18 (62.1)	0	8 (12.5)	0	0	28 (5.3)
*An. triannulatus* (*s.s*.)	8 (2.7)	0	0	0	0	0	0	0	8 (1.5)
*Chagasia bonneae*	0	1 (1.0)	0	0	0	10 (15.6)	5 (83.3)	4 (25.0)	20 (3.8)
Other anophelines	80 (27.1)	0	11 (44.0)	9	0	9 (14.1)	0	0	100 (18.8)
Subtotal	295	97	25	29	0	64	6	16	532
Total	1018	208	216	244	13	148	103	16	1966

*Abbreviations*: *OX* oxbow lakes, *NOX* non-oxbow lakes, *FAAR* flooded areas associated with the river, *FANAR* flooded areas not associated with the river, *RP* rainfall pools, *SFS* small forest streams, *MFS* medium forest streams, *RIV* rivers

*Anopheles oswaldoi* (*s.l*.) was collected from all types of larval habitats, except rainfall pools. *Anopheles triannulatus* (*s.s*.) was almost exclusively collected in OX and NOX larval habitats. *Anopheles mattogrossensis* was more abundant in larval types associated with the river flood pulses, such as OX, NOX and FAAR. *Anopheles guarao*-like was found a wide variety of hydrological types in the three Yanomami areas and *An. costai*-like was also collected from different larval habitats, although in this case, larvae were more abundant in water bodies that were not associated with river flood pulses, such as FANAR, SFS and MFS. *Chagasia bonneae* were collected almost exclusively from water bodies with some degree of water movement, such as SFS, MFS and margins of low-order rivers. The other anophelines section of Table 2 included less frequent species such as *An. nimbus*, *An. thomasi*, *An. kompi* and *An. squamifemur*, which were collected from shaded SFS and FANAR larval habitats, *An. intermedius* which we found in OX and FAAR hydrological type and *An. nuneztovari* (*s.l*.) which was only collected from sun-exposed OX and co-occurring with other *Nyssorhynchus* species such as *An. darlingi*, *An. oswaldoi* (*s.l*.) and *An. triannulatus* (*s.s*.)*.*

### Association of anopheline species with environmental factors

**Fig. 2:**
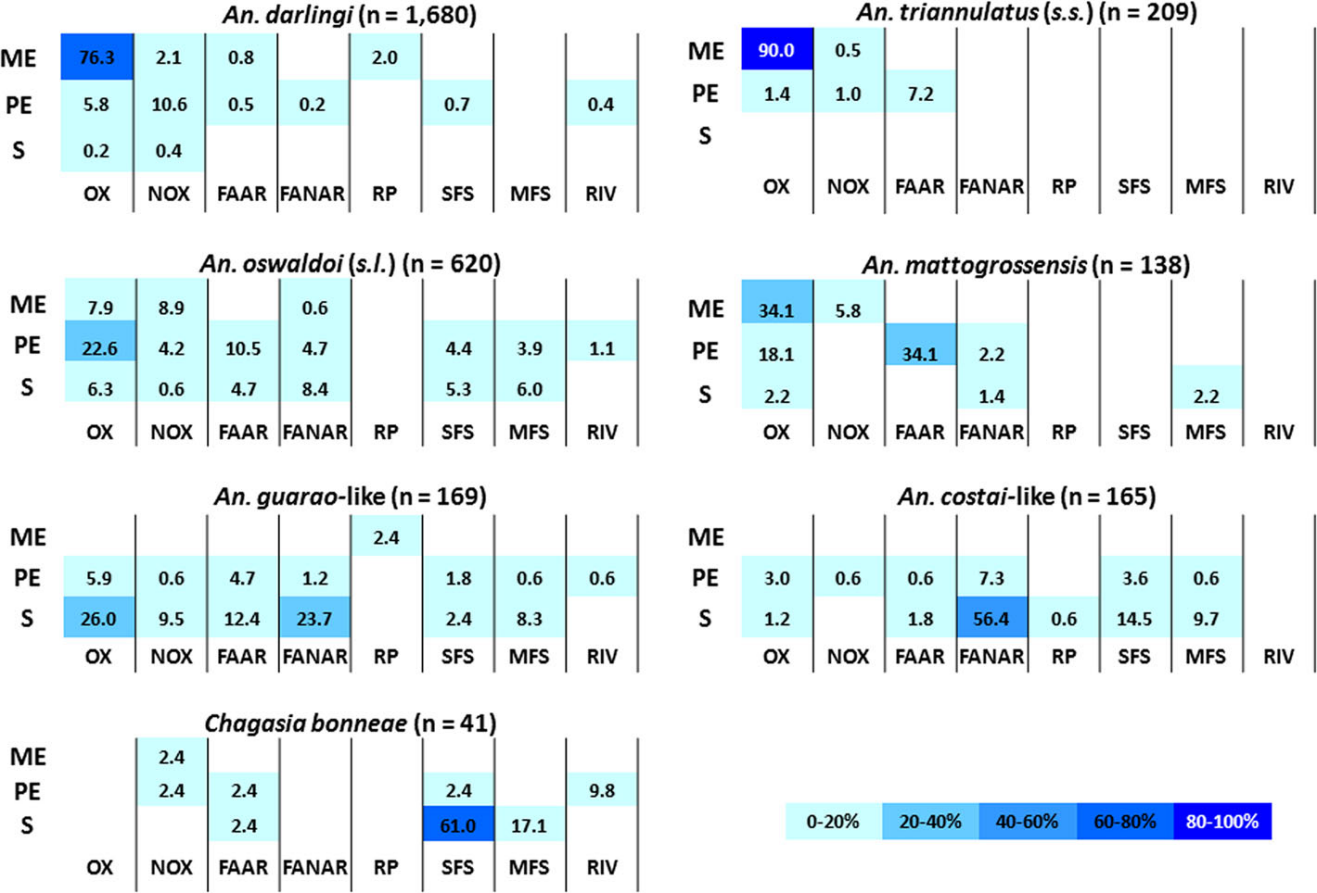
Multi-panel representation of the preference of each anopheline species (in %) for different combinations of larval habitat types and degree of sun exposure. *Abbreviations*: S, shaded; PE, partially exposed to the sun; ME, mostly exposed to the sun; OX, oxbow lakes; NOX, non-oxbow lakes; FAAR, flooded areas associated with the river; FANAR, flooded areas not associated with the river; RP, rainfall pools; SFS, small forest streams; MFS, medium forest streams; RIV, rivers. The total number of specimens of each species collected is given in parentheses. Note that for this figure, we have considered the sum of all larval instars for *An. darlingi*. Colors indicate the percentages of each species collected for each combination of hydrological type and degree of sun exposure

The data for mean number of each anopheline species adjusted for 100 dips and their associations with the seven environmental factors considered in our analysis are presented in Table 3. Figure 2 provides a schematic distribution displaying the associations of the seven species considered for analysis with different combinations of larval habitat hydrological types and degree of sun exposure. For example, 76.3% of all *An. darlingi* were collected from OX that were classified as sun-exposed.

**Table 3:** Mean number of larvae per 100 dips of each anopheline species for each environmental variable. The 95% confidence intervals are provided in parentheses

Variable	*An. darlingi*	*An. oswaldoi* (*s.l*.)	*An. triannulatus* (*s.s.*)	*An. mattogrossensis*	*An. costai-*like	*An. guarao-*like	*Chagasia bonnae*
Associated with flood pulses							
No (*n* = 361)	0.14 (0–0.35)	0.64 (0.36–0.92)	None	0.01 (0–0.03)	0.41 (0.25–0.56)	0.18 (0.11–0.25)	0.20 (0.002–0.42)
Yes (*n* = 350)	3.15 (1.37–4.94)	1.19 (0.78–1.61)	0.58 (0.17–0.98)	0.30 (0.17–0.43)	0.05 (0–0.12)	0.44 (0.05–0.83)	0.03 (0–0.06)
Seasonality							
Permanent (*n* = 435)	2.47 (1.03–3.91)	0.87 (0.56–1.17)	0.44 (0.12–0.77)	0.15 (0.07–0.24)	0.13 (0.04–0.23)	0.29 (0–0.58)	0.17 (0.002–0.35)
Seasonal (*n* = 276)	0.29 (0.01–0.58)	0.98 (0.56–1.40)	0.04 (0–0.08)	0.16 (0.05–0.26)	0.39 (0.23–0.56)	0.35 (0.15–0.55)	0.03 (0.006–0.06)
Sun exposure							
Shaded (*n* = 424)	0.01 (0–0.03)	0.46 (0.27–0.64)	None	0.01 (0–0.03)	0.31 (0.18–0.45)	0.31 (0.16–0.45)	0.18 (0.003–0.36)
Partially exposed (*n* = 200)	1.11 (0.41–1.82)	1.71 (1.05–2.37)	0.07 (0.003–0.14)	0.28 (0.11–0.45)	0.16 (0.02–0.30)	0.44 (0–1.06)	0.04 (0–0.09)
Mostly exposed (*n* = 87)	10.66 (3.75–17.57)	1.29 (0.34–2.25)	2.17 (0.56–3.77)	0.54 (0.18–0.91)	None	0.03 (0–0.10)	0.01 (0–0.3)
Turbidity							
Clear (*n* = 480)	1.70 (0.67–2.73)	0.50 (0.36–0.64)	0.37 (0.08–0.66)	0.11 (0.04–0.18)	0.30 (0.19–0.41)	0.32 (0.04–0.60)	0.18 (0.02–0.34)
Semi-turbid (*n* = 160)	1.86 (0–4.35)	1.33 (0.69–1.97)	0.13 (0–0.33)	0.28 (0.09–0.48)	0.04 (0–0.07)	0.34 (0.14–0.54)	None
Turbid (*n* = 71)	0.61 (0.18–1.04)	2.74 (0.99–4.49)	0.08 (0–0.21)	0.17 (0–0.38)	0.22 (0–0.65)	0.16 (0–0.33)	None
Water movement							
Yes (*n* = 176)	0.07 (0–0.15)	0.38 (0.19–0.57)	None	0.02 (0–0.05)	0.26 (0.11–0.42)	0.11 (0.02–0.19)	0.45 (0.02–0.87)
No (*n* = 535)	2.14 (0.96–3.32)	1.08 (0.76–1.41)	0.38 (0.11–0.64)	0.20 (0.11–0.29)	0.22 (0.12–0.33)	0.38 (0.12–0.64)	0.01 (0–0.03)
Submersed macrophytes							
Absent (*n* = 674)	0.67 (0.37–0.97)	0.94 (0.68–1.20)	0.03 (0.01–0.05)	0.15 (0.08–0.21)	0.24 (0.15–0.34)	0.33 (0.12–0.53)	0.13 (0.01–0.24)
Present (*n* = 37)	19.03 (3.16–34.90)	0.42 (0.03–0.81)	4.94 (1.28–8.60)	0.30 (0–0.67)	0.02 (0–0.06)	None	None

The outcomes of the multivariate analyses for the 6 *Anopheles* spp*.* and *Chagasia bonneae* are summarized in Table 4. For species in the subgenus *Nyssorhynchus*, we found that *An. darlingi* occurrence was positively associated with proximity to Yanomami dwellings (*Z* = -4.10, *P* < 0.0001), larval habitats associated with river flood pulses (*Z* = -2.29, *P* = 0.022), aquatic habitats that were partially or mostly exposed to the sun (*Z* = -6.37*, P* < 0.0001) and clear or semi-turbid waters over turbid ones (*Z* = 3.20, *P* = 0.001 and *Z* = 2.00, *P* < 0.046, respectively). However, the univariate analysis also detected positive associations of *An. darlingi* with permanent water bodies (*Z* = 2.47, *P =* 0.014), with stagnant waters (*Z* = -8.83, *P* < 0.0001) and with the presence of submerged macrophytes (*Z* = -4.43, *P* < 0.0001). *Anopheles oswaldoi* (*s.l*.) showed preference only for larval habitats which were partially and mostly sun-exposed (*Z* = -2.20*, P* = 0.028) and for semi-turbid and turbid larval habitats (*Z* = -3.55*, P* = 0.0004). The univariate analysis also detected significantly more *An. oswaldoi* (*s.l*.) in larval habitats without water movement (*Z* = -2.22, *P* = 0.026) and the absence of submersed macrophytes (*Z* = 2.34, *P* = 0.02). Larvae of *An. triannulatus* (*s.s*.) were significantly associated with permanent larval habitats (*Z* = 2.23, *P* = 0.026) and with the presence of submersed macrophytes (*Z* = -5.82, *P* < 0.0001). The univariate model for this species indicated that larvae was negatively associated with proximity to human habitations (*Z* = 2.10, *P* = 0.036) and preferred water bodies that were mostly exposed to the sun compared to partially exposed ones (*Z* = -5.04, *P* < 0.0001). No larvae of *An. triannulatus* (*s.s*.) were collected from shaded water bodies, larval habitats not associated with river flood pulses and with water movement.

**Table 4:** Risk ratios (lower 95% CL, upper 95% CL) from the adjusted (multivariate) regression models of the association of environmental factors with anopheline occurrence. Statistically significant (*P* < 0.05) outcomes are indicated with an “*”. In the case of three-level variables, statistically significant differences between the two non-reference categories are indicated with different letters within the same column. For the continuous variable “Distance to nearest hut” we report the risk ratio and the significance level (*P* < 0.05)

Variable	*An. darlingi*	*An. oswaldoi* (*s.l*.)	*An. triannulatus* (*s.s*.)	*An. mattogrossensis*	*An. costai*-like	*An. guarao*-like	*Chagasia bonneae*
Distance to nearest hut	0.99672*	1.00026	1.0004	1.00288*	0.9998	0.9993	0.997*
Associated with flood pulses							
No	0.22 (0.06–0.81)*	1.25 (0.58–2.70)	None	0.10 (0.02–0.46)*	12.08 (4.84–30.14)*	0.90 (0.33–2.41)	0.08 (0.02–0.26)*
Yes	Ref.	Ref.	All	Ref.	Ref.	Ref.	Ref.
Seasonality							
Permanent	2.83 (0.82–9.71)	0.66 (0.40–1.09)	19.25 (1.43–259.15)*	0.10 (0.03–0.33)*	0.55 (0.27–1.13)	0.74 (0.34–1.59)	1.93 (0.52–7.19)
Seasonal	Ref.	Ref.	Ref.	Ref.	Ref.	Ref.	Ref.
Sun exposure							
Shaded	0.006 (0.001–0.03)*^a^	0.36 (0.15–0.90)*^a^	None	0.01 (0.004–0.06)*^a^	1.33 (0.57–3.10)	12.11 (1.06–138.21)*^a^	1.80 (0.19–17.12)
Partially exposed	0.15 (0.06–0.37)*^b^	1.40 (0.66–2.95)^b^	3.01 (0.53–17.16)	0.10 (0.04–0.27)*^b^	1	6.83 (0.67–69.17)^a^	0.55 (0.04–8.02)
Mostly exposed	Ref.	Ref.	Ref.	Ref.	None	Ref.	Ref.
Turbidity							
Clear	5.34 (1.92–14.88)*^a^	0.29 (0.15–0.57)*^a^	0.43 (0.09–1.94)^a^	3.55 (1.15–10.95)*^a^	0.97 (0.14–6.50)^a^	1.18 (0.36–3.86)^a^	All
Semi-turbid	2.35 (1.02–5.45)*^a^	0.66 (0.33–1.30)^b^	0.32 (0.06–1.71)^a^	2.23 (0.9–5.48)^a^	0.21(0.03–1.65)^b^	2.29 (0.61–8.55)^a^	None
Turbid	Ref.	Ref.	Ref.	Ref.	Ref.	Ref.	None
Water movement							
Yes	0.36 (0.08–1.59)	0.70 (0.24–2.03)	None	5.91 (0.58–59.93)	0.52 (0.23–1.15)	0.42 (0.17–1.03)	200.3 (55.7–720.0)*
No	Ref.	Ref.	All	Ref.	Ref.	Ref.	Ref.
Submersed macrophytes							
Absent	0.96 (0.14–6.81)	1.74 (0.75–4.05)	0.02 (0.01–0.07)*	4.15 (1.01–17.05)*	1.01 (0.16–6.63)	All	All
Present	Ref.	Ref.	Ref.	Ref.	Ref.	None	None

*Abbreviations*: *All* all specimens collected in that category, *None* no specimens collected in that category

Within the members of the *Anopheles* subgenus, significantly more larvae of *An. mattogrossensis* were found in larval habitats further from the human habitations (*Z* = 3.54, *P* = 0.0004), associated with flood pulses (*Z* = -2.96*, P* = 0.003), seasonal water bodies (*Z* = -3.85*, P* = 0.0001), mostly and partially sun-exposed (*Z* = -5.97, *P* < 0.0001), clear waters over turbid ones (*Z* = 2.20, *P* = 0.028) and without submersed macrophytes (*Z* = 1.97, *P* = 0.048). In univariate analyses, *An. mattogrossensis* was also positively associated with larval habitats without water movement (*Z* = -2.07, *P* = 0.038), and positive associations concerning the adjusted analysis were only retained for the preference of larval habitats associated with flood pulses and sun exposure. In the adjusted model for *An. costai*-like, only a significant association with larval habitats out of the reach of river flood pulses was detected (*Z* = 5.34*, P* < 0.0001). However, when considering the univariate analysis, *An. costai*-like was also significantly more abundant in seasonal larval habitats (*Z* = -2.75, *P* = 0.006), shaded larval habitats (*Z* = 2.84, *P* = 0.005) and water bodies without the presence of submerged macrophytes (*Z* = 2.64, *P* = 0.008). For *An. guarao*-like, the only variable explaining the occurrence of this species was its preference for shaded over mostly sun-exposed larval habitats (*Z* = 2.01, *P* = 0.045). In the univariate analysis, only stagnant larval habitats were positively associated with more larvae (*Z* = -2.34, *P* = 0.019). This species was never found in larval habitats with the presence of submersed macrophytes.

Finally, *Chagasia bonneae* was positively associated with larval habitats closer to the Yanomami dwellings (*Z* = -2.18, *P* = 0.029), with river flood pulses (*Z* = -4.19, *P* < 0.0001) and with water movement (*Z* = 8.12, *P* < 0.0001). Nonetheless, contrarily to the adjusted model, the univariate analysis indicated positive associations with larval habitats not associated with river flood pulses (*Z* = 3.04*, P* = 0.002), permanent (*Z* = 2.14, *P* = 0.032) and shaded (*Z* = 2.48, *P* = 0.013). These results indicate a very strong modulating effect between variables for this species.

## Discussion

During a two-year larval collection in the Brazilian Amazon, within low-order river-floodplain systems (first to fifth river order), *An. darlingi* was the second most abundant species only exceeded in numbers by *An. oswaldoi* (*s.l*.). In contrast, larvae of *An. darlingi* have been regarded as difficult to find. In Belize for example, no *An. darlingi* larvae were found over a two-year study [30]. In the Zo’é Indian Reserve of Brazil, a total of 6392 adults of *An. darlingi* were collected while only 26 larvae were recorded during three field expeditions [31].

*Anopheles darlingi* has been found in many different larval habitats, natural [13, 30–34] and man-made [13, 35] as well as from large and permanent to small and temporary water bodies [13]. This species has been characterized by its adaptability to different and changing ecological environments [33]. Microdams, which are sections of streams and rivers where water surface flow is obstructed by overhanging twigs or fallen stumps coupled with the accumulation of floating debris have been reported as important larval habitats for *An. darlingi* [30, 32, 34]. In the Suriname Rainforest, *An. darlingi* was associated with seasonally flooded forest areas from the river and rain waters [36]. On the interior forested malaria-endemic area of Guyana, *An. darlingi* preferred waters found in forest streams, seepage swamps and larval rainwater habitats, while in the savannahs of the interior, lakes were reported as preferred larval habitats [37]. On the other hand, in the coastal areas of Guyana, *An. darlingi* larval habitats included man-made water bodies such as irrigation canals, rice fields and flooded drains and ditches [37].

Our results contrast with some of the previous observations of *An. darlingi* preference for certain larval habitats. For example, our immature anopheline collections were negative in the low-order rivers in the Toototobi and Parafuri areas. We believe this was because these river canals had no microenvironments suitable as anopheline larval habitats. These microenvironments would be represented by micro-dams within the 1 km upstream and downstream perimeter from each village. In the low-order rivers of Marari on the other hand, only a small number of anopheline larvae (including *An. darlingi*) were collected during the dry season, mainly in micro-dams exposed to sunlight created by fallen trees with floating debris, sunlight pools in the riverbed, and the edges of the river with emergent vegetation or filamentous algae. Nonetheless, although anopheline larvae were collected, low-order rivers were not considered primary anopheline larval habitats since these habitats, and the larvae collected, were present in low numbers. In Marari villages, LAR (and specially OX) within 1 km radius of each village constituted the main larval habitats for *An. darlingi*, *An. triannulatus* (*s.s*.), *An. oswaldoi* (*s.l*.) and *An. nuneztovari* (*s.l*.). OX were more frequently positive and had the highest densities of *An. darlingi* larvae compared to other hydrological types. Larval habitats similar to sun-exposed LAR were previously reported as primary larval habitats [23, 24, 38]. In the state of Bolivar in Venezuela, natural lagoons and artificial water bodies generated due to mining activities were considered the primary larval habitats for *An. darlingi* and *An. marajoara* [38]. Lagoons (which could correspond to OX, NOX or FAAR in our classification) were major larval habitats for *An. triannulatus* and *An. darlingi* in the Venezuelan Yanomami area [23, 24].

In our study sun exposure was a major determinant of anopheline occurrence. We encountered significantly more *An. darlingi* in larval habitats mostly (1365 larvae) or partially sun-exposed (305 larvae) compared with shaded ones (10 larvae). Our findings corroborate other reports on Neotropical anophelines. Galvão et al. [39] emphasized that exposure to the sun was a major factor that governed *An. darlingi* occurrence and reported that in shaded forest larval habitats, specimens of this vector were absent. However, if the same area suffered deforestation and exposed some parts of the previously fully shaded larval habitats to the sun, these new exposed spots became productive larval habitats for *An. darlingi* while adjacent shaded areas continued to be unsuitable for this species. Deane et al. [40] also reported few larvae of *An. darlingi* collected from shaded areas, and that vector preferred areas intensively exposed to the sun. Vittor et al. [41] conducted a larvae ecology study in the Amazon region of Peru. They found that larval habitats with < 70% of their water surface covered in the shade were nearly twice as likely to have *An. darling* than water bodies with > 70% of their surface covered in the shade. However, *An. darlingi* preference for shaded larval habitats were also observed [13]. In the Brazilian Amazon, shade seemed to be a major driver for *An. darlingi* proliferation in micro dam larval habitats [32] and fishponds [35] close to houses.

Remote indigenous communities in the Amazon are typically located in ecologically conserved areas where most of the anthropogenic impacts are in the form of subsistence agriculture near their huts where the forest is cut down and previously shaded larval habitats may be exposed to sunlight and potentially become larval habitats for *An. darlingi*. This was observed in small, ephemeral and sun-exposed RP in Parafuri and a sun-exposed SFS segment in Marari. In Marari besides micro deforestation for agriculture purposes, an airstrip construction created suitable habitats for *An. darlingi*, demonstrating the importance of small-scale landscape modifications. Forested fully shaded SFS and RP had no *An. darlingi* larvae during our study.

In addition to sunlight, the presence of certain subtypes of supportive EBA, such as submersed macrophytes, emergent vegetation, filamentous algae, water body margins with leafs or debris or clusters of floating debris inside LAR (further away from LAR’s shoreline) are necessary to support *An. darlingi* breeding. In the univariate analysis, both sunlight and submersed macrophytes were positively associated with *An. darlingi*. However, sunlight was the predominant factor in the multivariate analysis. The lack of significance for vegetation and *An. darlingi* presence in the multivariate analysis was likely due to collinearity between sun exposure and these other explanatory variables.

*Anopheles darlingi* larvae have been collected in areas with current water such as the edges of small rivers and canals [42, 43]. Although water movement was not a predictive factor in our multivariate model for *An. darlingi*, our unadjusted analysis indicated an association of this species with stagnant waters (conditions found in OX and NOX that disconnect seasonally from rivers). However, we also found a few *An. darlingi* larvae within streams and low-order rivers with low water movement. *Anopheles darlingi* is believed not to thrive in turbid or polluted waters [44]. Although we found significantly more *An. darlingi* larvae in clear waters, a few larvae were also collected in turbid LAR waters during its low-waters phase. *Anopheles darlingi* was previously reported in large, bare, and muddy road-pools [42].

Our data further indicated that *An. darlingi* was the only species in which densities were significantly higher in larval habitats closer to the Yanomami huts, suggesting a dependency on human blood. In a recent study in the Western Amazon, the same was observed with higher densities of *An. darlingi* in fishponds within 100 m of houses and an absence of malaria cases in places > 900 m from fishponds [45]. However, *An. darlingi* in the Lacandon forest of South México was found to be significantly more abundant in larval habitats away from human habitations [46].

Differences reported in the characteristics of *An. darlingi* larval habitats in the various studies might be due to *An. darlingi* genetic variability [46, 47] and local adaptation [48]. However, marked differences have also been reported in populations without clear biogeographical barriers such as our findings of an association of *An. darlingi* larvae with sun-exposed larval habitats and the shaded larval habitat preference reported in Southern Roraima state [32]. Different observations could also be due to how sun exposure is defined by different investigators as open pools surrounded by vegetation may experience some shading for certain parts of the day. Differences reported in the larval ecology of *An. darlingi* could also be due to differences in sampling methodology. Primary *An. darlingi* habitats such as OX which are relatively large and intrinsically complex pose serious sampling challenges, including accessibility for all EBA subtypes. Some authors have emphasized that in their studies, only accessible margins of water bodies were sampled for *An. darlingi* occurrence [35, 45, 49]. We found *An. darlingi* larvae mainly in OX and NOX hydrological types, which required the use of a small inflatable boat to access most EBA subtypes within the larval habitat. In fact, we found most *An. darlingi* larvae in EBA subtypes that were not accessible from the LAR perimeter (Sánchez-Ribas J, unpublished data). Different conclusions regarding the association between environmental factors and *An. darlingi* would have been obtained if we had not used the inflatable boat in our study. We and other authors [26, 48, 49] warn against the strategy of focusing the sampling on the margins of aquatic habitats and advocate for extending the collections to other EBA subtypes within larval habitats. To circumvent this problem, we have recently proposed a standardized sampling methodology, which may be applicable in size variable and intrinsically complex Neotropical larval sites [26].

*Anopheles oswaldoi* (*s.l*.) showed marked ecological plasticity being found in almost all hydrological types and all villages of the three Yanomami communities. *Anopheles oswaldoi* (*s.l*.) is a complex composed of at least three different species, i.e. *An. oswaldoi* (*s.s*.), *An. oswaldoi* A and *An. oswaldoi* B [50] that are widely distributed in South America and have been found in a wide range of larval habitats, with a marked tolerance of different degrees of sun exposure, turbidity and larval habitat sizes [19]. *Anopheles oswaldoi* (*s.l*.) was also the most common species collected as larvae, followed by *An. triannulatus* and *An. darlingi* during the dry season in partially shaded and shallow lagoons in the Yanomami area of Ocamo, Venezuela [24] and the nearby Ye’kuana and Sanema indigenous areas of southeastern Venezuela [25]. In a littoral area of the northeastern Sucre State of Venezuela, *An. oswaldoi* (*s.l*.) was primarily found in permanent and vegetated ponds of freshwater and non-vegetated canals [51]. *Anopheles oswaldoi* (*s.l*.) was associated with heavily shaded swamps in Panama [52]. Molecular analysis (cytochrome *c* oxidase subunit 1 gene, *cox*1) on adult mosquitoes detected the co-occurrence of *An. oswaldoi* B, *An. oswaldoi* sp. nr. A and a single specimen which did not match with any of this other two groups (Sánchez-Ribas J, unpublished data). High ecological plasticity detected for *An. oswaldoi* (*s.l*.) larvae could be partially explained by potential bionomic differences between members of this species complex that co-occur in the Brazilian Yanomami Indian Reserve.

Preferred larval habitats of *An. triannulatus* have been described as partial sun-exposed water bodies associated with emergent, submerged or floating macrophytes such as freshwater swamps, permanent ponds, lakes, ditches and river margins [53] but also in seasonal water bodies such as rock holes, small ground pools and animal tracks [44]. *Anopheles triannulatus* was classified as a habitat generalist in a study conducted in the Roraima and Pará States of Brazil, with a widespread local distribution without clear environmental associations [54]. In the Pantanal region of Brazil, the three sibling species of the *An. triannulatus* complex, *An. triannulatus* (*s.s*.), *An. halophylus* and *An. triannulatus* species C, were associated with large floodplain water bodies, most of them permanent [53]. Although *An. triannulatus* (*s.s*.) and *An. halophylus* exploited similar water bodies, they differed in their salinity tolerance, with the former species found in fresh waters and the latter in brackish water bodies [53]. *Anopheles triannulatus* (*s.s*.) is present in Central and South America while *An. halophylus* and *An. triannulatus* C has only been found in a geographically restricted area of central-western Brazil [55]. All specimens collected by us were identified as *An. triannulatus* (*s.s*.) and found almost exclusively in partially or mostly sun-exposed permanent larval habitats, without water movement and strongly associated with submerged macrophytes. Previous reports also showed *An. triannulatus* (*s.s*.) significantly associated with less shaded with submerged macrophytes lagoons in the Yanomami Venezuelan area of Ocamo [23] and also in Venezuela, *An. triannulatus* was found in dry season river bed pools and clusters of floating vegetation during the rainy season [56]. However, no *An. triannulatus* (*s.s*.) larvae were collected by us in SFS or MFS and river canal-related larval habitats.

Although *An. mattogrossensis* has been incriminated as a secondary malaria vector in Brazil [57], few data have been published regarding its bionomics, particularly on the ecology of its immature forms. *Anopheles mattogrossensis* is a species of the subgenus *Anopheles*, and its larval habitats were more similar to species of the subgenus *Nyssorynchus* such as *An. triannulatus* (*s.s*.) and *An. darlingi* rather than with the other species of its subgenus (e.g. *An. guarao-*like and *An. costai-*like). *Anopheles mattogrossensis* was more likely to be found in partially or mostly sun-exposed larval habitats. Specimens morphologically identified as *An.costai-*like were found in shaded larval habitats not associated with river flood pulses. Larval habitats exploited by *An. guarao-*like were similar to those of *An. oswadoi* (*s.l*.), being collected in all types of water bodies but with a preference for shaded water bodies. Even for the most generalist species found in our study area, *An. oswaldoi* (*s.s*.) and *An. guarao-*like, a significant predictor of larvae occurrence was sun exposure. The taxonomic status of *An. costai-*like and *An.guarao-*like remain unresolved, and therefore we were unable to compare our data with previously published studies. Work is underway to elucidate the formal identities of species classified as *An. costai-*like and *An. guarao-*like in the present publication.

Finally, *Chagasia bonneae* was associated with larval habitats that were predominantly shaded (univariate analysis), without submersed macrophytes and with some degree of water movement. These characteristics were found mainly in shaded SFS and MFS and also in shaded or partially exposed margins of low-order rivers. Filamentous algae were usually absent from SFS and MFS. In Venezuela, this species has been collected from stream edges with algae and partially exposed to the sun [58]. In the Zo’é Indian Reserve of Brazil, a dense Amazonian forested area traversed by the low-order Cuminipanema river, *Chagasia bonneae* was the most common species collected as immatures, although the preferred hydrological type of this species was not specified [31]. Another member of the genus, *Chagasia bathana,* was found in shaded running streams, mainly in areas of slowed current due to projected roots into the streams as well as within debris and dead leaves of side pools of streams [52].

Our study had a number of limitations that should be considered when interpreting the results. A major challenge for any quantitative study of the larval ecology of mosquitoes is the difficulty in reliably sampling larvae. For this study, a rigorous sampling methodology was followed in all larval habitat types and attempted to sample all EBA subtypes within the habitat, including those EBA distant from the shoreline of large larval habitats that are normally out of reach. Data such as physicochemical measures of temperature, pH, total dissolved salts (TDS), conductivity and dissolved oxygen were not included in the uni- and multivariate regression models since we were unable to measure them reliably during the whole study period. Additionally, our sun exposure classification did not take into account the amount of time that each segment of EBA subtype was exposed to direct sunlight. Also, we were unable to identify and consider for statistical analysis first and second instars for all species, except *An. darlingi*. Molecular taxonomic analyses were not conducted on larvae and several mosquitoes identified in this study are known to be complexes of species. Molecular studies to identify these mosquitoes are ongoing. Small samples sizes for *Chagasia bonneae* (only 41 specimens) may have led to spurious results, such as the observation that *Chagasia bonneae* densities were higher in larval habitats located closer to the Yanomami huts while only three adult specimens of *Chagasia bonneae* were sampled in all collections highlighting a low anthropophilic biting profile.

## Conclusions

We identified high heterogeneity in species composition and larval densities between Yanomami communities and villages. This variability was explained by the different availability of larval habitats with different intrinsic characteristics at a micro-scale level. We confirmed that LAR, and especially sun-exposed OX, were key for the maintenance of local malaria vector populations of *An. darlingi*, *An. triannulatus* (*s.s*.), *An. oswaldoi* (*s.l*.) and *An. mattogrossensis*. Sun exposure was a major modulator for the occurrence of the majority of anophelines. *Anopheles darlingi* thrived in those few spots which were exposed to sunlight within an overwhelmingly predominant shaded forest environment. In this study, natural larval habitats of *An. darlingi* in remote indigenous areas located within low-order river-floodplain systems represent an excellent opportunity to incorporate feasible and sustainable LSM approaches. Targeting immature anophelines may be a cost-effective intervention in some specific resilient malaria hotspots of remote indigenous areas of the Amazon basin and Central America. Finally, more information on the ecology of Neotropical anopheline immature forms is needed in other settings, particularly for *An. darlingi*, to better understand the key environmental drivers that may modulate the occurrence of the main malaria vector of the Amazon basin.

## Additional files

Additional file 1: Table S1. Total and percentage of late instar anopheline larvae collected per village. (XLSX 12 kb)

Additional file 2: Table S2. Dataset used for univariate and multivariate statistical analysis. (XLSX 263 kb)

## Abbreviations

FAAR: Flooded areas associated with the river
FANAR: Flooded areas not associated with the river
GPS: Global positioning system
IRS: Indoor residual spraying
LAR: Lakes associated with the river
LLINs: Long-lasting insecticidal nets
LSM: Larval source management
MFS: Medium forest streams
NOX: Non-oxbow lakes
OX: Oxbow lakes
RP: Rainfall pools
SFS: Small forest streams
tEBA: Total effective breeding area
